# Dissimilatory iodate-reducing microorganisms inhabit marine oxygen minimum zones

**DOI:** 10.1093/nsr/nwag397

**Published:** 2026-06-29

**Authors:** Junxia Li, Zhou Jiang, Xueyun Li, Wenjie Fang, Yongguang Jiang, Yidan Hu, Yiran Dong, Xianjun Xie, Liang Shi, Andreas Kappler, Yanxin Wang

**Affiliations:** State Key Laboratory of Geomicrobiology and Environmental Changes & School of Environmental Studies, China University of Geosciences, Wuhan 430078, China; MOE Key Laboratory of Groundwater Quality and Health, China University of Geosciences, Wuhan 430078, China; State Environmental Protection Key Laboratory of Source Apportionment and Control of Aquatic Pollution, Ministry of Ecology and Environment, China University of Geosciences, Wuhan 430078, China; State Key Laboratory of Geomicrobiology and Environmental Changes & School of Environmental Studies, China University of Geosciences, Wuhan 430078, China; MOE Key Laboratory of Groundwater Quality and Health, China University of Geosciences, Wuhan 430078, China; State Key Laboratory of Geomicrobiology and Environmental Changes & School of Environmental Studies, China University of Geosciences, Wuhan 430078, China; State Key Laboratory of Geomicrobiology and Environmental Changes & School of Environmental Studies, China University of Geosciences, Wuhan 430078, China; State Key Laboratory of Geomicrobiology and Environmental Changes & School of Environmental Studies, China University of Geosciences, Wuhan 430078, China; State Key Laboratory of Geomicrobiology and Environmental Changes & School of Environmental Studies, China University of Geosciences, Wuhan 430078, China; State Key Laboratory of Geomicrobiology and Environmental Changes & School of Environmental Studies, China University of Geosciences, Wuhan 430078, China; MOE Key Laboratory of Groundwater Quality and Health, China University of Geosciences, Wuhan 430078, China; Hubei Key Laboratory of Yangtze Catchment Environmental Aquatic Science, China University of Geosciences, Wuhan 430078, China; State Key Laboratory of Geomicrobiology and Environmental Changes & School of Environmental Studies, China University of Geosciences, Wuhan 430078, China; MOE Key Laboratory of Groundwater Quality and Health, China University of Geosciences, Wuhan 430078, China; State Environmental Protection Key Laboratory of Source Apportionment and Control of Aquatic Pollution, Ministry of Ecology and Environment, China University of Geosciences, Wuhan 430078, China; State Key Laboratory of Geomicrobiology and Environmental Changes & School of Environmental Studies, China University of Geosciences, Wuhan 430078, China; MOE Key Laboratory of Groundwater Quality and Health, China University of Geosciences, Wuhan 430078, China; Hubei Key Laboratory of Yangtze Catchment Environmental Aquatic Science, China University of Geosciences, Wuhan 430078, China; Geomicrobiology Group, Department of Geosciences, University of Tübingen, Tübingen 72074, Germany; State Key Laboratory of Geomicrobiology and Environmental Changes & School of Environmental Studies, China University of Geosciences, Wuhan 430078, China; MOE Key Laboratory of Groundwater Quality and Health, China University of Geosciences, Wuhan 430078, China; State Environmental Protection Key Laboratory of Source Apportionment and Control of Aquatic Pollution, Ministry of Ecology and Environment, China University of Geosciences, Wuhan 430078, China; Hubei Key Laboratory of Yangtze Catchment Environmental Aquatic Science, China University of Geosciences, Wuhan 430078, China

**Keywords:** iodine, marine oxygen minimum zones, dissimilatory iodate-reducing microorganisms, nitrate reduction

## Abstract

Based on theoretical thermodynamic calculations, microbial IO_3_^−^ reduction precedes NO_3_^−^ reduction, and it was previously proposed that dissimilatory iodate-reducing microorganisms (DIRMs) inhabit a unique niche above marine oxygen minimum zones (OMZs). Here we demonstrate that dissimilatory IO_3_^−^ reduction lags behind NO_3_^−^ reduction in two representative strains *Azonexus hydrophilus* NCP973 and *Denitromonas iodatirespirans* IR-12. Correspondingly, the functional genes *idrABP1P2* for DIRMs were found to be exclusively distributed across depth profiles of global OMZs where NO_3_^−^ reduction is active. Combined with widespread detection and heterologous expression of the *idrABP1P2* of metagenome-assembled genomes (MAGs) from the OMZs, these findings suggest that DIRMs inhabit marine OMZs and contribute to I^−^ production and accumulation. As OMZs expand under global warming, DIRMs could enhance volatile iodine fluxes to the atmosphere by producing the precursor I^−^. Given the environmental health importance of atmospheric iodine, integrating this pathway into marine iodine biogeochemical models will improve our capability of understanding and predicting the future changes in oceanic iodine emissions.

## Teaser

Dissimilatory iodate-reducing microorganisms mediate the reduction of iodate to iodide, yet their ecological niche in the oceans remains unclear. This work reveals that these microorganisms inhabit marine oxygen minimum zones, where they drive the production and accumulation of iodide.

## INTRODUCTION

As a life-essential trace element, iodine and its global biogeochemical cycling are of great significance for the environment and human health [1]. Iodate (IO_3_^−^) reduction to more mobile iodide (I^−^) is one of the most crucial steps in iodine cycling, which controls the transport of iodine between hydrosphere, atmosphere and biosphere [2–4]. In the oceans, the largest iodine reservoir on Earth, I^−^ derived from IO_3_^−^ reduction is oxidized by ozone (O_3_) or algae at the sea-air boundary to form volatile iodine (e.g. I_2_, HIO and CH_3_I) [5,6]. Volatile iodine entering the atmosphere not only causes O_3_ destruction, atmospheric mercury depletion, and the formation of ultrafine aerosol particles that help cloud condensation and offset radiative forcing, but also affects continental iodine status through wet and dry deposition [1,7,8]. Additionally, IO_3_^−^ reduction in aquifers triggers the release of iron mineral-bound iodine and the enrichment of I^−^ in groundwater, thus posing a severe threat to the health of millions of people worldwide [9].

Dissimilatory IO_3_^−^-reducing microorganisms (DIRMs) are bacteria that use IO_3_^−^ as the terminal electron acceptor for respiration and reduce it into I^−^. To date, five DIRMs were isolated from marine and groundwater environments, including *Pseudomonas* sp. SCT [10], *Denitromonas iodatirespirans* IR-12 [11], *Aromatoleum toluclasticum* TC-10 [12], *Azoarcus* sp. DN11 [13] and *Azonexus hydrophilus* NCP973 [14]. The IO_3_^−^ reductase (Idr) in DIRMs is composed of IdrA, IdrB, IdrP1 and IdrP2 subunits, of which IdrAB reduces IO_3_^−^ to HIO and H_2_O_2_, and IdrP_1_P_2_ detoxifies H_2_O_2_ to H_2_O, followed by the disproportionation of HIO to I^−^ and IO_3_^−^ [2,11]. Based on the results of thermodynamic calculations for seawater (Fig. S1A) and *idrA* abundance in the Tara Oceans metagenomic dataset, it was proposed that DIRMs inhabit a unique niche at the upper fringes of marine oxygen minimum zones (OMZs), between the zones of O_2_ and NO_3_^−^ reduction [11]. However, our findings in iodine-rich groundwater challenge the proposed spatial distribution pattern of DIRMs in the aquatic system of the Earth. In our recent work at the North China Plain (NCP), we detected the *idrA* gene and isolated *A. hydrophila* NCP973 from groundwater where NO_3_^−^ was depleted [14,15]. Furthermore, a negative correlation between I^−^ and NO_3_^−^ concentrations is widespread across high-iodine groundwater systems in China, including the NCP, Hetao Basin, and Datong Basin (Fig. S2). These observations imply that IO_3_^−^ reduction by DIRMs may not occur before NO_3_^−^ reduction, a scenario that contradicts with the results of thermodynamic calculations for high-iodine groundwater (Fig. S1B).

Marine OMZs exist in midwater depths (100–1000 m) of the oceans with extremely low O_2_ concentration (≤20 µM), and are known hotspots for diverse redox processes including IO_3_^−^ reduction, denitrification, anammox, sulfate reduction and sulfur oxidation [16–20]. I^−^ and NO_2_^−^, as products of microbial reduction, co-accumulate and develop distinct concentration maxima within OMZs worldwide, such as the eastern tropical North Pacific (ETNP) [19,21], eastern tropical South Pacific (ETSP) [22] and Arabian Sea [18,23]. To date, the mechanism responsible for I^−^ enrichment in OMZs remains elusive. With our finding in high-iodine groundwater where IO_3_^−^ reduction does not precede NO_3_^−^ reduction in mind, we hypothesize that DIRMs reside within the OMZs and contribute to I^−^ production and accumulation. However, whether DIRMs inhabit the OMZs and what their diversity and function are remain unknown.

To test our hypothesis of the inhabitation of DIRMs in the OMZs and their role in I^−^ enrichment, this study first selected two representative DIRMs isolated from groundwater and marine environments, *A. hydrophilus* NCP973 and *D. iodatirespirans* IR-12, to determine the reduction order of IO_3_^−^ and NO_3_^−^. Thereafter, integrated analyses of metagenomes, metatranscriptomes, metagenome-assembled genomes (MAGs) and heterologous expression of *idrABP1P2* of MAGs from OMZs were performed to reveal the distribution, diversity and function of DIRMs in global OMZs.

## RESULTS AND DISCUSSION

### Sequential reduction of NO_3_^−^ and IO_3_^−^ by *A. hydrophilus* NCP973 and *D. iodatirespirans* IR-12

**Figure 1.**
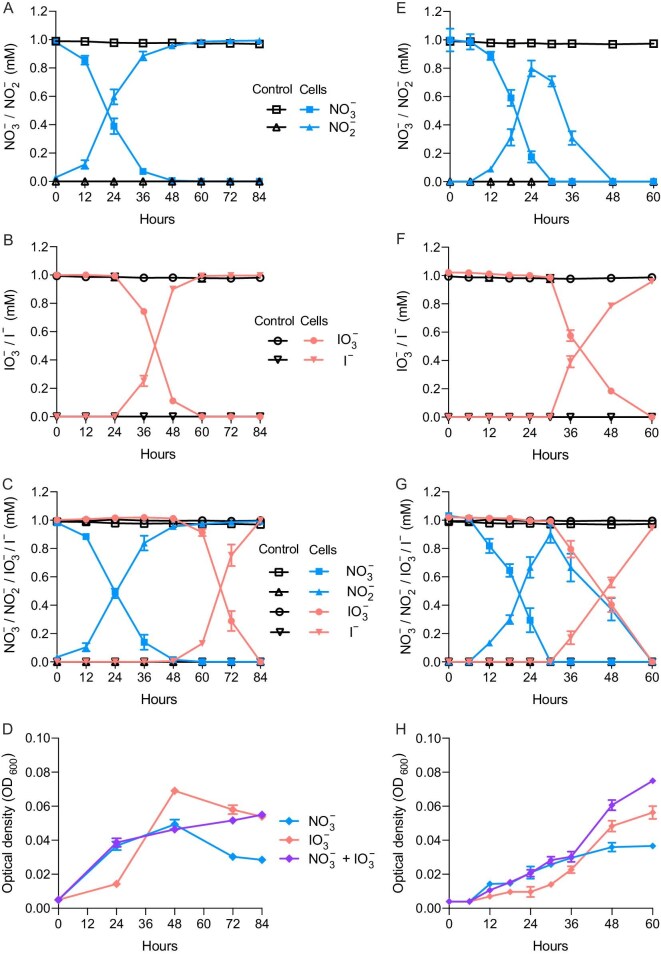
NO_3_^−^ and IO_3_^−^ reduction and cell growth of *A. hydrophilus* NCP973 (A–D) and *D. iodatirespirans* IR-12 (E–H). (A) and (E) denote the cultures containing 10 mM acetate and 1 mM NO_3_^−^. (B) and (F) denote the cultures containing 10 mM acetate and 1 mM IO_3_^−^. (C) and (G) denote the cultures containing 10 mM acetate, 1 mM NO_3_^−^ and 1 mM IO_3_^−^. (D) and (H) refer to cell growth under the above three culture conditions. Error bars represent standard deviation of triplicates (*N* = 3). For those without error bars, the error bars are smaller than the size of the symbols.

To determine the order of reducing NO_3_^−^ and IO_3_^−^, *A. hydrophilus* NCP973 and *D. iodatirespirans* IR-12 were cultured in the presence of 10 mM acetate (electron donor) and 1 mM NO_3_^−^, IO_3_^−^, or both as electron acceptors. As shown in Fig. 1, in the presence of 1 mM NO_3_^−^ or IO_3_^−^, *A. hydrophilus* NCP973 and *D. iodatirespirans* IR-12 were able to completely reduce NO_3_^−^ to NO_2_^−^ or IO_3_^−^ to I^−^, respectively. This is consistent with the presence of NO_3_^−^-reducing genes *napAB*/*narGHI* and IO_3_^−^-reducing genes *idrABP1P2* in their genomes (Fig. S3). Different from *A. hydrophilus* NCP973, *D. iodatirespirans* IR-12 can further perform NO_2_^−^ reduction via denitrification pathway (Fig. S3B), as evidenced by the detection of N_2_O during the late stage of incubation (Fig. S4). Compared with NO_3_^−^ reduction, IO_3_^−^ reduction by both strains had a ∼24 h longer lag phase. Microbial IO_3_^−^ reduction produces more energy than NO_3_^−^ reduction to NO_2_^−^ at the same molar concentration of the acceptor (Fig. S1). The difference in energetics of redox reactions was evidenced by the observation that the OD_600_ values of the cultures of both strains were higher after complete IO_3_^−^ reduction than after complete NO_3_^−^ reduction (Fig. 1D and H). Thermodynamically, the reduction of IO_3_^−^ by DIRMs precedes the reduction of NO_3_^−^ when these two electron acceptors co-occur [11,24]. However, in the presence of both 1 mM NO_3_^−^ and 1 mM IO_3_^−^, *A. hydrophilus* NCP973 and *D. iodatirespirans* IR-12 first reduced NO_3_^−^ to NO_2_^−^ and then reduced IO_3_^−^ to I^−^ (Fig. 1C and G). This stepwise reduction of NO_3_^−^ and IO_3_^−^ was also observed in cultures of both strains containing 1 mM NO_3_^−^ and 0.1 mM IO_3_^−^ (Fig. S5). To date, all five isolated DIRMs harbor NO_3_^−^ reductase genes (*napAB*/*narGHI*) in their genomes, and can grow with NO_3_^−^ as a sole electron acceptor [10–14]. Given the ability of these five DIRMs to reduce both NO_3_^−^ and IO_3_^−^ and the results of our experiments using two of the five DIRMs, it is reasonable to infer that IO_3_^−^ reduction by DIRMs lags behind NO_3_^−^ reduction, which is different from DIRMs' niche in natural environments based on thermodynamic calculations (Fig. S1).

The transcriptomic results of these two strains in the presence of both 1 mM NO_3_^−^ and 1 mM IO_3_^−^ showed that *narGHI* and *idrABP1P2* were significantly upregulated in the stages of NO_3_^−^ reduction and IO_3_^−^ reduction, respectively (Fig. S6). Although *idrABP1P2* of *D. iodatirespirans* IR-12 was upregulated in the stage of NO_3_^−^ reduction, its expression level was 16-fold lower than that in the following stage of IO_3_^−^ reduction (Fig. S6). Therefore, in the presence of two electron acceptors, *narGHI* and *idrABP1P2* were expressed sequentially in both strains and mediated the stepwise reduction of NO_3_^−^ and IO_3_^−^ observed in Fig. 1C and G.

The pattern that IO_3_^−^ reduction by these two DIRMs lags behind NO_3_^−^ reduction in the presence of both NO_3_^−^ and IO_3_^−^ is similar to the lag of (per)chlorate reduction behind NO_3_^−^ reduction observed in dissimilatory (per)chlorate-reducing bacteria, soil microcosms, and biofilm reactors [25–28]. The lag of selenate reduction behind NO_3_^−^ reduction was also reported in field sample microcosms and biofilm reactors, although the selenium-respiring bacteria *Sulfurospirillum barnesii* and *Thauera selenatis* can reduce NO_3_^−^ and selenate simultaneously [29–31]. In this study, the sequential reduction of NO_3_^−^ and IO_3_^−^ by DIRMs, which differs from thermodynamic predictions, could be attributed to NO_3_^−^-mediated suppression of *idrABP1P2* expression and IO_3_^−^-induced oxidative stress. As shown in Fig. S6, the *idrABP1P2* genes of *A. hydrophilus* NCP973 was not significantly upregulated during the stage of NO_3_^−^ reduction. In contrast, although the *idrABP1P2* genes of *D. iodatirespirans* IR-12 showed low-level upregulation during NO_3_^−^ reduction, this strain was unable to reduce IO_3_^−^ to I^−^ at that stage. The reason why these two DIRMs prefer NO_3_^−^ over IO_3_^−^ is likely related to the lower energy barriers for proton and electron transfers associated with NO_3_^−^ reduction [26,27]. Similarly, NO_3_^−^ was reported to negatively regulate (per)chlorate reductase and chlorite dismutase expression and repress (per)chlorate reduction in *Dechlorosoma suillum* strain PS and *Dechlorosoma* sp. KJ [25,32]. As an oxidizing agent, IO_3_^−^ impacts cell growth and metabolism through oxidative stress and even leads to cell death, especially at elevated concentrations [33,34]. Our previous study showed that the growth of *A. hydrophilus* NCP973 was negatively affected when the amount of added IO_3_^−^ exceeded 4 mM [14]. Given the feature of the lag phase for the repair and replacement of damaged subcellular components [35], IO_3_^−^-induced oxidative stress resulted in a prolonged lag phase for *A. hydrophilus* NCP973 and *D. iodatirespirans* IR-12 to initiate IO_3_^−^ reduction (Fig. 1B and F). In contrast, the shorter lag phase under NO_3_^−^ reduction may confer a competitive advantage to both strains, thus enabling them to reduce NO_3_^−^ before IO_3_^−^ in the presence of dual electron acceptors. This physiological characteristic of DIRMs would constrain their distribution in natural environments [24]. Collectively, these results indicate that IO_3_^−^ reduction by DIRMs lags behind NO_3_^−^ reduction, highlighting the need to understand the distribution of DIRMs in the environments from both thermodynamic and physiological perspectives.

### Distribution and diversity of DIRMs in marine OMZs

**Figure 2.**
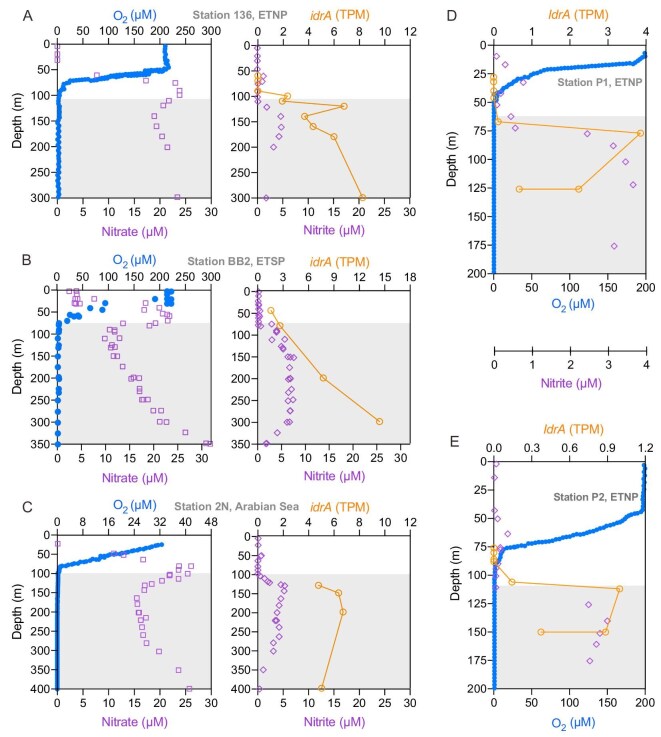
Depth profiles of *idrA* gene (A–C) and transcript (D, E) abundances as well as O_2_, NO_3_^−^, and NO_2_^−^ concentrations at the ETNP, ETSP and Arabian Sea sampling stations. O_2_, NO_3_^−^ and NO_2_^−^ concentrations at the station 136 (ETNP), BB2 (ETSP), 2N (Arabian Sea), P1 and P2 (ETNP) were from Fuchsman *et al.* [36], Sun and Ward [37], Fortin *et al.* [38] and Mattes *et al.* [39], respectively. (D) and (E) share the same NO_2_^−^ concentration coordinate between them. The abundances of *idrA* gene and transcript were retrieved from seawater metagenomes and metatranscriptomes at these sampling stations (Table S1), respectively. The OMZ area at each station is highlighted with a gray background.

The lag of IO_3_^−^ reduction by DIRMs behind NO_3_^−^ reduction implies that DIRMs reduce IO_3_^−^ to I^−^ only when NO_3_^−^ reduction has already taken place. Following this finding, we propose that DIRMs should inhabit a unique niche within marine OMZs characterized by NO_3_^−^ reduction, rather than above the OMZs as previously suggested [11]. Therefore, it may not be a coincidence that the *idrA* gene was indeed detected at high abundance only in seawater metagenomes from depth profiles of OMZs in the ETNP, ETSP and Arabian Sea (Fig. 2A–C and Table S1). Furthermore, metatranscriptome analysis of seawater from two depth profiles in the ETNP indicated that high levels of *idrA* transcript were found only in their OMZ (Fig. 2D, E). The OMZ of onshore station P1 contained a higher *idrA* transcript abundance than that of offshore station P2, suggesting that the input of nutrients from natural and anthropogenic sources favors the growth and reproduction of DIRMs. The detection of *idrA* in both metagenomics and metatranscriptomics from global OMZs indirectly indicates the presence and activity of DIRMs in marine OMZs, although future studies involving DIRMs isolation, *in situ* incubation and direct rate measurements to verify and evaluate their ecological contribution are still needed. These multi-omics data from this study support previous predictions about the potential contribution of DIRMs to IO_3_^−^ reduction in the OMZs based on the oxygen tolerance of IdrABP1P2 [40]. The observations of high NO_2_^−^ concentrations and high abundances of the *idrA* gene and transcript in the three major OMZs (Fig. 2) further suggest that NO_3_^−^ reduction occurs before IO_3_^−^ reduction by DIRMs.

**Figure 3.**
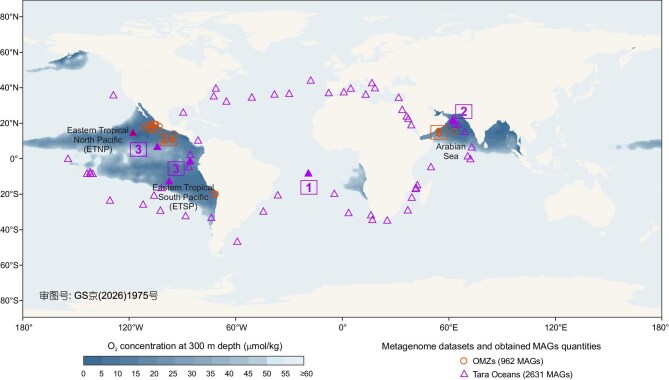
Distribution of *idrABP1P2*-containing MAGs from marine OMZs (**○**) and Tara oceans metagenomes (△). Each site has multiple metagenome samples at different depths, and 962 and 2631 MAGs were obtained from OMZs [41] and Tara oceans metagenomes [42], respectively. The *idrABP1P2* was identified among these MAGs, and the number of MAGs containing *idrABP1P2* is shown in the box next to their parent metagenomes. Tara Oceans metagenomes containing MAGs with *idrABP1P2* are highlighted with solid purple triangles. Dissolved O_2_ concentrations in the water column are taken from the World Ocean Atlas 2023.

To determine the diversity of DIRMs in marine OMZs, we analyzed two MAGs datasets obtained from global OMZs and oceans (Tara Oceans Expedition), respectively. The results showed that 32 of 962 MAGs from global OMZs contain *idrABP1P2*, and were mainly distributed in OMZs of the ETNP and Arabian Sea (Fig. 3). Comparatively, 234 metagenomic samples collected from 61 stations at multiple depths during Tara Oceans Expedition generated 2631 MAGs [42], of which nine MAGs were found to harbor *idrABP1P2* (Fig. 3). Notably, these nine MAGs with *idrABP1P2* were all from samples in the mesopelagic zone of the ETNP, ETSP and Arabian Sea. The geographic distribution of MAGs containing *idrABP1P2* in this study was similar to that of *idrA* detected in Tara Oceans metagenome samples [11]. The absence of *idrABP1P2-*containing MAGs in the surface water layer and deep chlorophyll maximum layer as well as in non-OMZs worldwide further support that DIRMs occupy a unique ecological niche in marine OMZs.

**Figure 4.**
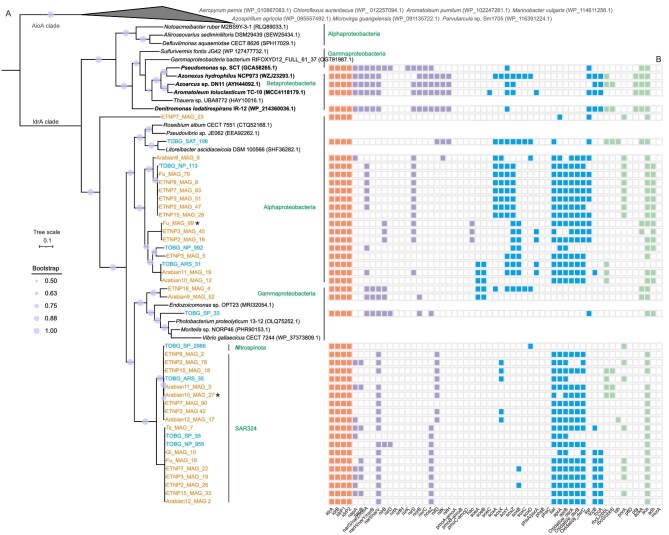
The phylogeny of IdrA in MAGs obtained from marine OMZs (A) and heatmap of key functional genes of iodine (orange), nitrogen (purple), sulfur (blue) and carbon metabolisms (green) in these MAGs (B). The AioA serve as the outgroup and are collapsed. Reference sequences of AioA (gray nodes) and IdrA (black nodes) refer to Reyes-Umana *et al.* [11], with IdrA of the five isolated DIRMs highlighted in bold. Orange and blue nodes denote MAGs from the OMZ dataset [41] and Tara Oceans dataset [42], respectively. Two MAGs whose *idrABP1P2* functions were validated by heterologous expression are marked with black stars. Detailed information on IdrA sequences and corresponding MAGs is provided in Table S2. In the right heatmap, solid and hollow squares indicate the presence and absence of the corresponding functional genes in MAGs, respectively.

The 41 *idrABP1P2*-containing MAGs identified in this study exhibit completeness ranging from 56.90% to 98.91% (mean: 88.00%) and contamination ranging from 0% to 6.90% (mean: 2.02%) (Table S2), which greatly expand the diversity of DIRMs known to date. The phylogenetic tree of IdrA showed that the IdrA of MAGs from marine OMZs forms a new clade, which is distinct from the IdrA of isolated DIRMs within the class *Betaproteobacteria*/*Gammaproteobacteria* (Fig. 4A). This different clade of IdrA with isolated DIRMs was also observed in the IdrA phylogeny from seawater metagenomes and metatranscriptomes of global OMZs (Fig. S7). Based on the taxonomy of MAGs (Table S2), the IdrA of MAGs from marine OMZs were mainly classified as candidate phylum SAR324 (46.3%) and the class *Alphaproteobacteria* (43.9%), which is different from widespread *Betaproteobacteria*-affiliated IdrA in iodine-rich groundwater metagenomes worldwide [14]. This result indicates that DIRMs inhabiting global marine OMZs may be novel representative species capable of IO_3_^−^ reduction.

### Functional characterization of *idrABP1P2*-containing MAGs in marine OMZs

To validate their IO_3_^−^ reduction abilities, two *idrABP1P2* sequences of Fu_MAG_99 from *Alphaproteobacteria* and Arabian10_MAG_27 from SAR324 (Fig. 4A) were synthesized and expressed in *Escherichia coli* DH5α. As shown in Fig. S8, both *E. coli* DH5α strains with cloned *idrABP1P2* gene cluster showed significantly higher IO_3_^−^ reduction extents during 96 h of incubation in the medium containing 10 mM acetate and 0.24 mM IO_3_^−^ than the control *E. coli* DH5α with an empty vector. The latter showed a ∼28% IO_3_^−^ reduction, and no IO_3_^−^ reduction was observed in the cell-free control and heat-killed cells control, which are similar to the results of our previous studies [14,15]. The partial IO_3_^−^ reduction by the empty-vector control further suggests that *E. coli* DH5α harbors either a novel IO_3_^−^-specific reductase or a nonspecific reductase with IO_3_^−^ reducing capability. The amount of IO_3_^−^ consumed was stoichiometrically balanced with the amount of I^−^ generated. These results imply that these novel *idrABP1P2* in MAGs obtained from the OMZs encode functional enzymes that reduce IO_3_^−^ to I^−^.

Different from isolated DIRMs harboring genes for almost complete denitrification pathway (*napAB, narGHI, nirS, norBC* and *nosZ*), these IO_3_^−^-reducing MAGs obtained from the OMZs were characterized by the presence of sulfur oxidation pathways (Fig. 4B). We found that 73.2% of these MAGs contain key functional genes of complete dissimilatory sulfide oxidation pathway, such as oxidative *dsrABC, aprAB* and *sat*, especially in the candidate phylum SAR324 and in the class *Alphaproteobacteria* (Fig. 4B). Additionally, thiosulfate oxidation genes (*soxABCDXYZ*) and sulfide oxidation genes (*fccAB, sqr*) were detected in some of these MAGs and isolated DIRMs. Previous studies have documented a cryptic sulfur cycle and sulfide-driven denitrification in the OMZs [16,43,44]. The co-occurrence of *idrABP1P2* and sulfur-oxidizing genes in MAGs from global OMZs suggests that sulfide oxidation may be coupled to IO_3_^−^ reduction, as we proposed in high-iodine groundwater of the Hetao Basin in China [45]. Sulfide-oxidizing IO_3_^−^-reducing bacteria in the OMZs may be either chemolithoautotrophic or chemolithoheterotrophic, because three MAGs (Arabian10_MAG_27, Arabian11_MAG_3 and ETNP15_MAG_18) in the candidate phylum SAR324 contain the key carbon-fixing gene *cbbLS* encoding ribulose-1,5-bisphosphate carboxylase/oxygenase (Fig. 4B). This is consistent with metapangenomic results showing that the SAR324 lineage from the dark or twilight oceans has a sulfur-based chemolithoautotrophic lifestyle [46]. Given the ubiquitous distribution of SAR324 in environments [47], we further collected all representative SAR324 MAGs from the GTDB to detect *idrABP1P2* occurrence across diverse habitats (Table S3). As shown in Fig. S9, the *idrABP1P2*-containing MAGs in SAR324 are found only in seawater with low-oxygen concentrations, especially in the OMZs. The biogeochemical conditions favorable for both active sulfur and iodine cycling in the OMZs may provide a unique ecological niche for these sulfide-oxidizing IO_3_^−^-reducing bacteria in SAR324.

Collectively, these results suggest that DIRMs in the OMZs couple IO_3_^−^ reduction to oxidation of organic matter or reduced sulfur species. In contrast to previously proposed pathways of IO_3_^−^ reduction including abiotic reduction by iron(II)/reduced sulfur species and biotic reduction by iron(III)/sulfate-reducing bacteria [48–50], the dissimilatory IO_3_^−^ reduction mediated by DIRMs identified herein offers a more plausible mechanism for I^−^ enrichment in global OMZs, such as the ETNP [19,21], ETSP [22] and Arabian Sea [18,23].

### Environmental implications of DIRMs activities in marine OMZs

Global warming reduces O_2_ solubility in seawater and weakens ventilation owing to thermal stratification of the water column, thus leading to ocean deoxygenation. As a direct consequence, marine OMZs expand in geographic distribution and have even lower oxygen concentrations in their centers [17,51]. It was estimated that the area of OMZs in the global oceans had increased from 5% to 14% over the past 60 years, and would continue to rise substantially in the future [52]. Additionally, increased nutrient loading from anthropogenic sources causes coastal and estuarine eutrophication which can drive the formation and exacerbation of hypoxic zones [53]. As these oxygen-depleted zones expand and intensify, DIRMs are expected to have larger habitats and their activities reduce more IO_3_^−^ in seawater to I^−^. Since the oceans are the largest iodine reservoir, the reduction of IO_3_^−^ to I^−^ by DIRMs as well as other organisms in the oceans play vital roles in global iodine cycling, with I^−^ acting as the oxidation substrate by O_3_ or algae to produce volatile iodine (e.g. I_2_, HIO and CH_3_I) at the sea-air boundary [5,6]. The more I^−^ is produced in seawater, the more volatile iodine will be released into the atmosphere. Ice core data from East Greenland [54] and the French Alps [55] show that the iodine levels in the atmosphere increased 3-fold from 1950 to 2010, primarily due to the enhanced I^−^ production in seawater and anthropogenic O_3_ pollution.

Elevated atmospheric iodine levels have important environmental and health implications. On one hand, the atmospheric iodine enhances the destruction of tropospheric O_3_, the depletion of gaseous elemental mercury, and the formation of aerosols and cloud condensation, all of which have significant impacts on air quality and climate change [7,8,56,57]. On the other hand, the deposition of gaseous iodine onto the continents offers a long-term potential of increasing global iodine status, which either promotes thyroidal health of human living in iodine-deficient regions or exacerbates thyroidal dysfunction among residents exposed to excessive iodine intake [1,9,58]. Because of the significance of atmospheric iodine, efforts to parameterize sea-surface I^−^ fluxes and integrate them into models have been made to improve the understanding of future changes in oceanic iodine emissions [8,59–61]. Modeling results show that a 1% increase in global sea-surface I^−^ concentration induces a ∼0.7% rise in oceanic iodine emissions [5]. Given the kinetical stability of I^−^ [50,62], the I^−^ produced by DIRMs in OMZs can be transported to surface seawater through ocean circulation. Thus, IO_3_^−^ reduction by DIRMs in OMZs revealed from this study should be integrated into ocean iodine biogeochemical cycling models to better predict future changes in surface ocean I^−^ concentration and oceanic iodine emissions.

## METHODS

### Thermodynamic calculations

The Gibbs free energy (*ΔG_r_*) of acetate oxidation coupled to O_2_, IO_3_^−^, or NO_3_^−^ reduction at environmentally relevant conditions (seawater and high-iodine groundwater) was calculated using:


\begin{eqnarray*}
\Delta {{G}_r} = \left( {\Delta G_r^0 + \mathit{RTln}\left[ {\prod {{a}_i}^{{{v}_i}}} \right]} \right)/z,
\end{eqnarray*}

where the definitions and calculation procedures of the symbols are described in the Supplementary Materials.

### Strain, media and culture conditions

*Azonexus hydrophilus* NCP973 and *D. iodatirespirans* IR-12 were precultured anaerobically at 30°C in chemically defined medium (CDM) [13] and artificial pore water medium (APM) [10], respectively, with 10 mM acetate and 1 mM IO₃⁻ as electron donor and acceptor. Strain sources, media preparation, and anoxic handling refer to the Supplementary Materials.

### NO_3_^−^ and IO_3_^−^ reductions and transcriptomic analysis

To determine the reduction order of IO_3_^−^ and NO_3_^−^, *A. hydrophilus* NCP973 and *D. iodatirespirans* IR-12 were inoculated in CDM and APM with 10 mM acetate and either 1 mM NO_3_^−^, 1 mM IO_3_^−^, or both (1 mM each), respectively. Given the lower environmentally relevant concentrations of IO_3_^−^, we also tested cultures with 10 mM acetate, 1 mM NO_3_^−^ and 0.1 mM IO_3_^−^. Uninoculated media served as abiotic controls. All treatments were incubated in triplicate at 30°C under anoxic conditions. Concentrations of IO_3_^−^, I^−^, NO_3_^−^, NO_2_^−^ and N_2_O and the optical density at 600 nm (OD₆₀₀) were measured as detailed in the Supplementary Materials.

To detect *napAB*/*narGHI* and *idrABP1P2* expression during stages of NO_3_^−^ and IO_3_^−^ reduction in cultures with both NO_3_^−^ and IO_3_^−^, cells were harvested for RNA extraction and sequencing at 0, 24, and 72 h (*A. hydrophilus* in CDM, Fig. 1C) and at 0, 24, and 48 h (*D. iodatirespirans* in APM, Fig. 1G). RNA extraction, sequencing, and subsequent data analyses are provided in the Supplementary Materials.

### Vertical abundance of *idrA* gene and transcript in global marine OMZs

A total of 18 representative seawater metageno-mes from three OMZ profiles in the ETNP, ETSP and Arabian Sea and 16 representative seawater metatranscriptomes from two OMZ profiles in the ETNP were downloaded from the NCBI SRA database (Table S1). Raw metagenomic and metatranscriptomic reads were processed, annotated, and used to determine *idrA* abundance (Supplementary Materials).

### Diversity and function of *idrABP1P2*-containing MAGs in the oceans

Two MAGs datasets [962 MAGs from global OMZs and 2631 MAGs from global oceans (Tara Oceans Expedition)] were downloaded from the NCBI genome database [41,42]. MAGs harboring *idrABP1P2* were identified and subsequently analyzed for their diversity and function potential (Supplementary Materials).

### Heterologous expression of *idrABP1P2* of MAGs from OMZs

Two *idrABP1P2* DNA sequences from representative OMZs MAGs were synthesized and cloned into *Escherichia coli* DH5α to validate IO_3_^−^-reducing ability. Detailed experimental procedures are described in the Supplementary Materials.

### Identification of *idrABP1P2* in candidate phylum SAR324

A total of 335 representative SAR324 MAGs were downloaded from the GTDB (v10.RS226) to detect the presence of *idrABP1P2* (Table S3). The identification of *idrABP1P2* in these MAGs followed the same approach as described above for the marine MAGs. A treemap was generated in R using the WeightedTreemaps package to illustrate the distribution of SAR324 MAGs containing *idrABP1P2* in natural environments.

## Supplementary Material

nwag397_Supplemental_Files

## Data Availability

The transcriptomic sequencing raw data of *A. hydrophilus* NCP973 and *D. iodatirespirans* IR-12 have been submitted to NCBI’s SRA database under accession number PRJNA1336414. The datasets of metagenome, metatranscriptome and MAGs were download directly from the SRA and genome databases of NCBI, respectively, as detailed in Tables S1 and S2.
