# Supplementary material for: Dissimilatory iodate-reducing microorganisms inhabit marine oxygen minimum zones
Source: Natl Sci Rev. 2026 Jun 29;13(15):nwag397. doi: 10.1093/nsr/nwag397 (PMC13455634; doi:10.1093/nsr/nwag397)
Supplement: Supplementary file 1 [file nwag397_supplemental_files.zip › Supplementary Materials.docx]

Supplementary Materials for

**Dissimilatory iodate-reducing microorganisms inhabit marine oxygen minimum zones**

Junxia Li^1,2,3‡^, Zhou Jiang^1,2‡^, Xueyun Li^1^, Wenjie Fang^1^, Yongguang Jiang^1^, Yidan Hu^1^, Yiran Dong^1,2,4^, Xianjun Xie^1,2,3^, Liang Shi^1,2,4^, Andreas Kappler^5^, Yanxin Wang^1,2,3,4^*

^1^ State Key Laboratory of Geomicrobiology and Environmental Changes & School of Environmental Studies, China University of Geosciences, Wuhan 430078, China

^2^ MOE Key Laboratory of Groundwater Quality and Health, China University of Geosciences, Wuhan 430078, China

^3^ State Environmental Protection Key Laboratory of Source Apportionment and Control of Aquatic Pollution, Ministry of Ecology and Environment, China University of Geosciences, Wuhan 430078, China

^4^ Hubei Key Laboratory of Yangtze Catchment Environmental Aquatic Science, China University of Geosciences, Wuhan 430078, China

^5^ Geomicrobiology Group, Department of Geosciences, University of Tübingen, Tübingen 72074, Germany

^‡^These authors contributed equally to this work.

*Corresponding author. Mailing address: 388 Lumo Road, Wuhan 430078, China. Phone: +86(027)67883152. E-mail: yx.wang@cug.edu.cn

**The PDF file includes:**

Detailed description of the methods

Figs. S1 to S9

Tables S1, S4

Table S2 (separate file)

Table S3 (separate file)

**Thermodynamic calculations**

The Gibbs free energy (*Δ*G_r_) of a redox reaction was calculated using:

$$\Delta G_{r}=(\Delta G_{r}^{0}+RTln\left[ \prod{a_{i}}^{v_{i}} \right])/z$$

where*Δ*G_r_^0^ refers to the standard Gibbs free energy of reaction, given in kJ mol^−1^; *R* and *T* are the universal gas constant (8.314 J K^−1^mol^−1^) and the temperature in K (298.15 K), respectively; *a_i_* is the activity of species i; *v_i_* is the stoichiometric coefficient of species i; and *z* is the transferred electron number in the reaction. *Δ*G_r_^0^ values were calculated from the values of the standard Gibbs free energy of formation (*Δ*G_f_^0^) of reactants and products (Table S4). The *a_i_* was calculated using:

$$a_{r}=r_{i}m_{i}$$

where *r_i_* and *m_i_* are the activity coefficient and concentration of species i. Under seawater (*I* = 0.7 M) and high-iodine groundwater (*I* = 0.01 M) conditions, the activity coefficient was calculated using the “WATEQ” DEBYE-HÜCKEL equation, and extended DEBYE-HÜCKEL limiting-law equation, respectively [1]. Inorganic carbon speciation in aqueous phase across a range of pH was determined from the pH, and pK_a1_ (6.35) and pK_a2_ (10.33) under the standard temperature of 298.15 K.

**Strain, media and culture conditions**

*Azonexus hydrophilus* NCP973 (CCTCC AB2024179) was isolated from the deep high-iodine groundwater in the NCP [2]. *Denitromonas* *iodatirespirans* IR-12 (ATCC TSD-242) was purchased from American Type Culture Collection (Manassas, USA) [3]. The chemically defined medium (CDM) contains the following per liter: 0.225 g K_2_HPO_4_, 0.225 g KH_2_PO_4_, 0.46 g NaCl, 0.225 g (NH_4_)_2_SO_4_, 0.117 g MgSO_4_·7H_2_O, 4.2 g NaHCO_3_, 1 g yeast extract, 1 mL trace element solution, and 10 mL vitamin solution [2]. The artificial pore water medium (APM) contains the following per liter: 30.8 g NaCl, 1.0 g NH_4_Cl, 0.77 g KCl, 0.1 g KH_2_PO_4_, 0.20 g MgSO_4_·7H_2_O, 0.02 g CaCl_2_·2H_2_O, and 7.16 g HEPES, along with vitamin and mineral mixes as described in [3]. The pH of CDM and APM was adjusted to 8.0 and 7.2, respectively. The medium in serum bottles was boiled and purged with 100% N_2_ gas to remove dissolved oxygen, and then sealed with butyl rubber stoppers and aluminum caps. After autoclaving at 121 °C for 20 min, each liter of APM was amended with 34.24 mL 0.4 M CaCl_2_ and 26.07 mL 2 M MgCl_2_·6H_2_O from sterile stock solutions. Additionally, acetate (10 mM) and IO_3_^−^ (1 mM) were added to CDM and APM from sterile stock solutions as the electron donor and electron acceptor, respectively. All chemicals used in this study were purchased from Shanghai Macklin Biochemical Technology Co., Ltd (Shanghai, China) and Sinopharm International Co., Ltd (Shanghai, China).

**NO_3_^−^ and IO_3_^−^ reductions and transcriptomic analysis**

At the predetermined time intervals, concentrations of IO_3_^−^, I^−^, NO_3_^−^ and NO_2_^−^ and the optical density at 600 nm (OD₆₀₀) in reaction solutions were measured using ion chromatography (ECO IC, Metrohm AG, Switzerland) and UV-Visible spectrophotometer (GENESYS 50, Thermo Fisher Scientific, USA), respectively. Headspace gas samples from *D.* *iodatirespirans* IR-12 cultures containing 1 mM NO_3_^−^ as the sole electron acceptor were collected, and the N_2_O concentrations were determined using a gas chromatograph (Agilent 8890 GC-system, Agilent Technologies, USA).

RNA was extracted using the HiPure Bacterial RNA Kit (Magen, China). The mRNA sequencing library was constructed with the NEBNext® Ultra II Directional RNA Library Prep Kit for Illumina (New England Biolabs, USA) following manufacturer’s recommendations, and then sequenced on the Illumina NovaSeq 6000 platform. The mRNA sequencing library was constructed with the NEBNext® Ultra II Directional RNA Library Prep Kit for Illumina (New England Biolabs, USA) following manufacturer’s recommendations, and then sequenced on the Illumina NovaSeq 6000 platform. Raw reads were trimmed with Fastp (v0.23.0), and then mapped to NCBI Rfam database using Bowtie2 (v2.4.5) to remove rRNA reads. The filtered reads were aligned to respective isolate genomes using Bowtie2 (v2.4.5). RSEM (v1.3.3) was used to generate read counts and functional information for each gene. To assess gene expression levels at different stages of culture, transcripts per kilobase million (TPM) for each gene was calculated. Differentially expressed genes between two different reduction stages of cultures were analyzed using DESeq2 (v1.34.0). The P value was adjusted using Benjamini and Hochberg’s approach to determine the false discovery rate (FDR). Transcripts with FDR≤0.05 and |log_2_(Fold Change)|≥1 were defined as differentially expressed genes.

**Vertical abundance of *idrA* gene and transcript in global marine OMZs**

Raw metagenomic and metatranscriptomic reads were trimmed to generate clean data using Trimmomatic (v0.39) [4]. tRNA and rRNA sequences in metatranscriptomic clean reads were removed using SortMeRna (v4.3.6) [5]. The metaSPAdes (v3.15.0) [6] and trinity (v2.14.0) [7] were used to de novo assemble clean reads of metagenomics and metatranscriptomics into contigs, respectively. Open reading frames (ORFs) were predicted using Prodigal’s metagenome mode (v2.6.3) [8] and quantified using Salmon (v1.3.0) [9]. The ORFs were annotated by HMMSEARCH (v3.3.1) against KOfam (a customized Hidden Markov model (HMM) database of KEGG Orthologs) (-e 1e-5) [10]. The IdrA candidates was first identified from ORFs using the IdrA profile-HMM with a suggested threshold score of 640 [3]. Given the close relationship between IdrA and arsenite oxidase alpha subunit (AioA) [3,11], the AioA sequences in KEGG annotation results were also retrieved as IdrA candidates. At last, all IdrA candidates were validated by a maximum-likelihood phylogenetic tree of IdrA constructed using FastTree (v2.1.11) [2]. The abundance of IdrA‑positive sequences was extracted from the overall gene abundance table. For all software or tools used in this study, default parameters were chosen unless otherwise specified.

**Diversity and function of *idrABP1P2*-containing MAGs in the oceans**

The two downloaded MAGs datasets from global OMZs and oceans met a quality criterion of >50% completeness and <10% contamination [12,13]. The ORFs from MAGs were predicted using Prodigal and annotated by HMMSEARCH against KOfam (-e 1e-5). The screening and validation of IdrA in MAGs were the same as those for IdrA in metagenomics described above. Furthermore, other Idr subunits, such as IdrB, IdrP1 and IdrP2, were manually detected in the neighborhood of IdrA of MAGs. Only MAGs containing the *idrABP1P2* genes were subjected to downstream analysis. A maximum-likelihood phylogenetic tree of *idrA* was constructed using FastTree (v2.1.11) to illustrate the diversity of *idrA* in MAGs from global marine OMZs. Given that KEGG-annotated dissimilatory sulfite reductases (Dsr) function in either sulfite reduction or sulfide oxidation, we used another sequence-based Dsr classification tool (DiSCo) to determine the reductive or oxidative *dsrAB* from the ORFs [14]. At last, a heatmap was drawn using TBtools [15] to illustrate functional potential of MAGs containing *idrABP1P2*.

**Heterologous expression of *idrABP1P2* of MAGs from OMZs**

To experimentally validate IO_3_^−^-reducing ability of *idrABP1P2* detected, we synthesized two DNA sequences of *idrABP1P2* in representative MAGs from the OMZs. The synthesized DNA fragment and plasmid pBBR1MCS-2 were digested with restriction enzymes HindIII and Xba I (Thermo Scientific, Waltham, MA, USA), and then ligated with T4 DNA ligase (Thermo Scientific, Waltham, MA, USA) overnight at 16 ℃. The recombinant plasmids (pBBR1MCS-2-*idrABP1P2*) and empty vector (pBBR1MCS-2) were transformed into *Escherichia coli* DH5α. The positive clones of *E. coli* DH5α containing pBBR1MCS-2-*idrABP1P2* were verified by DNA sequencing. IO_3_^−^ reduction by *E. coli* DH5α with pBBR1MCS-2 and pBBR1MCS-2-*idrABP1P2* were conducted anaerobically in CDM with bacterial OD_600_ of 0.1, 0.24 mM IO_3_^−^ and 10 mM acetate at 37 ℃. Experiments without bacterial cell or with heat‑killed cells were set as controls. All treatments were performed in triplicate. At predetermined time intervals, the amount of IO_3_^−^ in reaction solutions was determined using the IO_3_^−^-triiodide method [2,16]. After 96 h of incubation, the concentrations of generated I^−^ were measured by ion chromatography (ECO IC, Metrohm AG, Switzerland). The *t*-tests were used to evaluate significance of differences in I^−^ content produced between strains containing *idrABP1P2* vector and empty vector.

Fig. S1. Gibbs free energy (ΔG_r_) of acetate oxidation coupled to oxygen (O_2_), iodate (IO_3_^−^), or nitrate (NO_3_^−^) reduction across a range of pH in seawater (A) and high-iodine groundwater (B) environments. Seawater conditions: ionic strength (*I*) = 0.7 M, T = 25 °C, $\mathbf{C}_{{\mathbf{C}\mathbf{H}_{\mathbf{3}}\mathbf{COO}}^{\mathbf{-}}}$ = 80 µM, C_DIC_ (dissolved inorganic carbon) = 2 mM, $\mathbf{C}_{\mathbf{IO}_{\mathbf{3}}^{\mathbf{-}}}$ = 0.45 µM, $\mathbf{C}_{\mathbf{I}^{\mathbf{-}}}$ = 0.01 µM, $\mathbf{C}_{\mathbf{NO}_{\mathbf{3}}^{\mathbf{-}}}$ = 10 µM, $\mathbf{C}_{\mathbf{NO}_{\mathbf{2}}^{\mathbf{-}}}$ = 0.1 µM, $\mathbf{C}_{\mathbf{O}_{\mathbf{2}}\mathbf{(aq)}}$ = 100 µM; high-iodine groundwater conditions: ionic strength (*I*) = 0.01 M, T = 25 °C, $\mathbf{C}_{{\mathbf{C}\mathbf{H}_{\mathbf{3}}\mathbf{COO}}^{\mathbf{-}}}$ = 100 µM, C_DIC_ = 10 mM, $\mathbf{C}_{\mathbf{IO}_{\mathbf{3}}^{\mathbf{-}}}$ = 0.01 µM, $\mathbf{C}_{\mathbf{I}^{\mathbf{-}}}$ = 10 µM, $\mathbf{C}_{\mathbf{NO}_{\mathbf{3}}^{\mathbf{-}}}$ = 1 µM, $\mathbf{C}_{\mathbf{NO}_{\mathbf{2}}^{\mathbf{-}}}$ = 1 µM, $\mathbf{C}_{\mathbf{O}_{\mathbf{2}}\mathbf{(aq)}}$ = 0.1 µM.

Fig. S2. Correlation between I^−^ and NO_3_^−^ concentrations in high-iodine groundwater in the Hetao Basin (A), Datong Basin (B), and North China Plain (C). The dataset of concentrations of I^−^ and NO_3_^−^ in high-iodine groundwater were obtained from our previous case studies (66-68).

Fig. S3. Key genes related to C, N, S and I metabolisms in the genomes of *Azonexus hydrophilus* NCP973 (A) and *Denitromonas iodatirespirans* IR-12 (B). Dashed arrows indicate the absence of key gene mediating the reactions.

Fig. S4. N_2_O concentrations detected during NO_3_^−^ reduction of *D.* *iodatirespirans* IR-12 in Fig. 1E. Error bars represent standard deviation of triplicates (N=3).

Fig. S5. NO_3_^−^ and IO_3_^−^ reduction by *A. hydrophilus* NCP973 (A) and *D.* *iodatirespirans* IR-12 (B) in cultures containing 10 mM acetate, 1 mM NO_3_^−^ and 0.1 mM IO_3_^−^ as well as I^−^ concentration generated after 48 h of incubation (C). Error bars represent standard deviation of triplicates (N=3). For those without error bars, the error bars are smaller than the size of the symbols.

**Fig. S6.** Expression of *napAB*/*narGHI* and *idrABP1P2* of *A. hydrophilus* NCP973 (A-B) and *D.* *iodatirespirans* IR-12 (C-D) at different stages of culture in medium containing 10 mM acetate, 1 mM NO_3_^−^ and 1 mM IO_3_^−^. A and C denote the NO_3_^−^ reduction stage in Fig. 1C and 1G. B and D denote the IO_3_^−^ reduction stage in Fig. 1C and 1G.

**Fig. S7.** The phylogeny of IdrA in seawater metagenomes and metatranscriptomes at the stations from the ETNP, ETSP and Arabian Sea. The AioA serves as the outgroup. Reference sequences (Black nodes) of AioA and IdrA refer to Reyes-Umana et al. (11), with IdrA of the five isolated DIRMs highlighted in bold. Blue, green and orange nodes denote the IdrA from OMZ seawater metagenomes, while red node denote the IdrA from OMZ seawater metatranscriptomes. Detailed description on metagenomes and metatranscriptomes and corresponding stations is provided in Table S1. ETNP: Eastern Tropical North Pacific Ocean; ETSP: Eastern Tropical South Pacific Ocean.

**Fig. S8.** IO_3_^−^ reduction of the *idrABP1P2* of Fu_MAG_99 and Arabian10_MAG_27 cloned in *E. coli* DH5α in CDM containing 10 mM acetate and 0.24 mM IO_3_^−^ (A) as well as the concentration of I^−^ generated after 96 h of incubation (B). Error bars represent standard deviation of triplicates (N=3). For those without error bars, the error bars are smaller than the size of the symbols. Significance levels: 0.01 < p < 0.05 (*), 0.001 < p < 0.01 (**), p < 0.001(***).

**Fig. S9.** Distribution of SAR324 MAGs with *idrABP1P2* (orange font)/without *idrABP1P2* (black font) across different marine and terrestrial environments in the GTDB database (v10.RS226). The number of *idrABP1P2*-containing MAGs associated with corresponding environment is highlighted in orange. Detailed information on SAR324 MAGs is provided in Table S3.

Table S1. Description of seawater metagenomes and metatranscriptomes at the stations from the ETNP, ETSP and Arabian Sea as well as the abundance of *idrA* gene and transcript.

| Ocean | Station ID | Coordinate | Accession Number | Depth (m) | Reference | *idrA* abundance (TPM) |
| --- | --- | --- | --- | --- | --- | --- |
| Metagenome |  |  |  |  |  |  |
| ETNP | 136 | 106.54°W, 17.04°N | SRR4465037 | 60 | Fuchsman et al. ^28^ | 0 |
| ETNP | 136 | 106.54°W, 17.04°N | SRR4465034 | 70 | Fuchsman et al. ^28^ | 0 |
| ETNP | 136 | 106.54°W, 17.04°N | SRR4465025 | 90 | Fuchsman et al. ^28^ | 0 |
| ETNP | 136 | 106.54°W, 17.04°N | SRR4465024 | 100 | Fuchsman et al. ^28^ | 2.348503 |
| ETNP | 136 | 106.54°W, 17.04°N | SRR4465027 | 110 | Fuchsman et al. ^28^ | 1.926168 |
| ETNP | 136 | 106.54°W, 17.04°N | SRR4465026 | 120 | Fuchsman et al. ^28^ | 6.795153 |
| ETNP | 136 | 106.54°W, 17.04°N | SRR4465029 | 140 | Fuchsman et al. ^28^ | 3.688057 |
| ETNP | 136 | 106.54°W, 17.04°N | SRR4465028 | 160 | Fuchsman et al. ^28^ | 4.354897 |
| ETNP | 136 | 106.54°W, 17.04°N | SRR4465036 | 180 | Fuchsman et al. ^28^ | 5.995349 |
| ETNP | 136 | 106.54°W, 17.04°N | SRR4465035 | 300 | Fuchsman et al. ^28^ | 8.251625 |
| ETSP | BB2 | 70.70°W, 20.50°S | SRR14610252 | 45 | Sun and Ward ^29^ | 1.573663 |
| ETSP | BB2 | 70.70°W, 20.50°S | SRR14610253 | 80 | Sun and Ward ^29^ | 2.631222 |
| ETSP | BB2 | 70.70°W, 20.50°S | SRR14610254 | 200 | Sun and Ward ^29^ | 7.750884 |
| ETSP | BB2 | 70.70°W, 20.50°S | SRR14610255 | 300 | Sun and Ward ^29^ | 14.397709 |
| Arabian Sea | 2N | 64.00°E, 15.15°N | SRR25584963 | 130 | Fortin et al. ^30^ | 4.777818 |
| Arabian Sea | 2N | 64.00°E, 15.15°N | SRR25584962 | 150 | Fortin et al. ^30^ | 6.362107 |
| Arabian Sea | 2N | 64.00°E, 15.15°N | SRR25584951 | 200 | Fortin et al. ^30^ | 6.716249 |
| Arabian Sea | 2N | 64.00°E, 15.15°N | SRR25584943 | 400 | Fortin et al. ^30^ | 5.028992 |
| Metatranscriptome |  |  |  |  |  |  |
| ETNP | P1 | 106.00°W, 20.15°N | SRR14460580 | 28 | Mattes et al. ^31^ | 0 |
| ETNP | P1 | 106.00°W, 20.15°N | SRR14460581 | 32 | Mattes et al. ^31^ | 0 |
| ETNP | P1 | 106.00°W, 20.15°N | SRR14460585 | 40 | Mattes et al. ^31^ | 0.00712 |
| ETNP | P1 | 106.00°W, 20.15°N | SRR14460572 | 46 | Mattes et al. ^31^ | 0.007036 |
| ETNP | P1 | 106.00°W, 20.15°N | SRR14460583 | 67 | Mattes et al. ^31^ | 0.123237 |
| ETNP | P1 | 106.00°W, 20.15°N | SRR14460573 | 77 | Mattes et al. ^31^ | 3.861583 |
| ETNP | P1 | 106.00°W, 20.15°N | SRR14460582 | 126 | Mattes et al. ^31^ | 2.239234 |
| ETNP | P1 | 106.00°W, 20.15°N | SRR14460584 | 126 | Mattes et al. ^31^ | 0.676538 |
| ETNP | P2 | 107.00°W, 16.90°N | SRR14460587 | 76 | Mattes et al. ^31^ | 0 |
| ETNP | P2 | 107.00°W, 16.90°N | SRR14460574 | 80 | Mattes et al. ^31^ | 0 |
| ETNP | P2 | 107.00°W, 16.90°N | SRR14460579 | 81 | Mattes et al. ^31^ | 0 |
| ETNP | P2 | 107.00°W, 16.90°N | SRR14460586 | 86 | Mattes et al. ^31^ | 0 |
| ETNP | P2 | 107.00°W, 16.90°N | SRR14460577 | 106 | Mattes et al. ^31^ | 0.143773 |
| ETNP | P2 | 107.00°W, 16.90°N | SRR14460576 | 112 | Mattes et al. ^31^ | 0.997537 |
| ETNP | P2 | 107.00°W, 16.90°N | SRR14460575 | 150 | Mattes et al. ^31^ | 0.885929 |
| ETNP | P2 | 107.00°W, 16.90°N | SRR14460578 | 150 | Mattes et al. ^31^ | 0.373832 |
| ETNP: Eastern Tropical North Pacific Ocean; ETSP: Eastern Tropical South Pacific Ocean | | | | | | |

Table S2. (separate file)

Summary of *idrABP1P2*-containing MAGs from marine OMZs and their IdrA sequences.

Table S3. (separate file)

Description of SAR324 genomes from the GTDB database (v10.RS226) and the presence/absence of *idrABP1P2* gene cluster in each genome.

Table S4. Standard Gibbs energy of formation (*Δ*G_f_^0^) and activity coefficient (γ) used for thermodynamic calculations.

| Compound | Formula | *Δ*G_f_^0^ (kJ mol-1)*^a^* | γ (*I* = 0.7 M)*^b^* | γ (*I* = 0.01 M)*^c^* | γ (*I* = 0.05 M)*^c^* |
| --- | --- | --- | --- | --- | --- |
| Iodate | IO_3_^-^ | -128 | 0.646 | 0.903 | 0.821 |
| Iodide | I^-^ | -51.57 | 0.627 | 0.898 | 0.808 |
| Acetate ion | CH_3_COO^−^ | -369.4 | 0.753 | 0.903 | 0.821 |
| Nitrate | NO_3_^-^ | -111.3 | 0.606 | 0.898 | 0.808 |
| Nitrite | NO_2_^-^ | -37.2 | 0.606 | 0.898 | 0.808 |
| Carbon dioxide | CO_2_ (aq) | -394.4 | -- | -- | -- |
| Oxygen | O_2_ (aq) | 16.32 | -- | -- | -- |
| Water | H_2_O | -137.13 | -- | -- | -- |
| *a*. Data source: Patrick L. Brezonik, William A. Arnold, Patrick L. Brezonik and William A. Arnold (2022) Water Chemistry: The Chemical Processes and Composition of Natural and Engineered Aquatic Systems, p. 0, Oxford University Press. *b*. Under *I* = 0.7 conditions corresponds to seawater, the activity coefficient was calculated using WATEQ” DEBYE-HÜCKEL equation. *c*. Under *I* = 0.01 and 0.05 conditions correspond to high iodine groundwater and incubation laboratory ecosystem, the activity coefficient was calculated using extended DEBYE-HÜCKEL limiting-law equation. | | | | | |

**References**

1.	Merkel B, Planer-Friedrich B, Nordstrom D. *Groundwater Geochemistry: A Practical Guide to Modeling of Natural and Contaminated Aquatic Systems*. Springer, 2008.

2.	Li J, Fang W, Li C *et al.* Dissimilatory iodate-reducing microorganisms contribute to the enrichment of iodine in groundwater. *Environ Sci Technol* 2024; **58**: 19255-19265.

3.	Reyes-Umana V, Henning Z, Lee K *et al.* Genetic and phylogenetic analysis of dissimilatory iodate-reducing bacteria identifies potential niches across the world's oceans. *ISME J* 2022; **16**: 38-49.

4.	Bolger AM, Lohse M, Usadel B. Trimmomatic: a flexible trimmer for Illumina sequence data. *Bioinformatics* 2014; **30**: 2114-2120.

5.	Kopylova E, Noé L, Touzet H. SortMeRNA: fast and accurate filtering of ribosomal RNAs in metatranscriptomic data. *Bioinformatics* 2012; **28**: 3211-3217.

6.	Nurk S, Meleshko D, Korobeynikov A *et al*. metaSPAdes: a new versatile metagenomic assembler. *Genome Res* 2017; **27**: 824-834.

7.	Grabherr MG, Haas BJ, Yassour M *et al.* Full-length transcriptome assembly from RNA-Seq data without a reference genome. *Nat Biotechnol* 2011; **29**: 644-652.

8.	Hyatt D, LoCascio PF, Hauser LJ *et al*. Gene and translation initiation site prediction in metagenomic sequences. *Bioinformatics* 2012; **28**: 2223-2230.

9.	Patro R, Duggal G, Love MI *et al*. Salmon provides fast and bias-aware quantification of transcript expression. *Nat Methods* 2017; **14**: 417-419.

10.	Aramaki T, Blanc-Mathieu R, Endo H *et al*. KofamKOALA: KEGG Ortholog assignment based on profile HMM and adaptive score threshold. *Bioinformatics* 2020; **36**: 2251-2252.

11.	Yamazaki C, Kashiwa S, Horiuchi A *et al*. A novel dimethylsulfoxide reductase family of molybdenum enzyme, Idr, is involved in iodate respiration by *Pseudomonas* sp. SCT. *Environ Microbiol* 2020; **22**: 2196-2212.

12.	Zhang IH, Sun X, Jayakumar A *et al*. Partitioning of the denitrification pathway and other nitrite metabolisms within global oxygen deficient zones. *ISME Commun* 2023; **3**: 76.

13.	Tully BJ, Graham ED, Heidelberg JF. The reconstruction of 2,631 draft metagenome-assembled genomes from the global oceans. *Sci Data* 2018; **5**: 170203.

14.	Boeuf D, Eppley JM, Mende DR *et al*. Metapangenomics reveals depth-dependent shifts in metabolic potential for the ubiquitous marine bacterial SAR324 lineage. *Microbiome* 2021; **9**: 172.

15.	Chen C, Chen H, Zhang Y *et al*. TBtools: An integrative toolkit developed for interactive analyses of big biological data. *Mol Plant* 2020; **13**: 1194-1202.

16.	Afkhami A, Madrakian T, Zarei AR. Spectrophotometric determination of periodate, iodate and bromate mixtures based on their reaction with iodide. *Anal Sci* 2001; **17**, 1199-1202.
